# Fetal Vibroacoustic Stimulation in Computerized Cardiotocographic Analysis: The Role of Short-Term Variability and Approximate Entropy

**DOI:** 10.1155/2012/814987

**Published:** 2012-01-16

**Authors:** Maria Laura Annunziata, Mariamaddalena Scala, Natascia Giuliano, Salvatore Tagliaferri, Olga Carmela Maria Imperato, Francesca Giovanna Esposito, Marta Campanile, Andrea Di Lieto

**Affiliations:** Department of Obstetrical-Gynaecological and Urological Science and Reproductive Medicine, Prenatal Care Unit, University “Federico II” of Naples, 80131 Naples, Italy

## Abstract

The aim of this study was to evaluate the impact of vibroacoustic stimulation (VAS) on computerized cardiotocography short-term variability (STV) and approximate entropy (ApEn) in both low- and high-risk pregnancies. VAS was performed on 121 high- and 95 low-risk pregnancies after 10 minutes of continuous quiet, while their FHR parameters were monitored and recorded by cCTG analysis. Fetal heart rate was recorded using a computer-assisted equipment. Baseline FHR, accelerations, decelerations, STV, long-term irregularity (LTI), ApEn, and fetal movements (FMs) were calculated for defined observational periods before VAS and after 10 minutes. Data were also investigated in relationship with the perinatal outcome. In each group of patients, FHR after VAS remained almost unmodified. Fetal movements significantly increased after VAS in both groups. Results show that only in the high-risk pregnancies, the increase of STV and the decrease of ApEn after VAS were significantly associated with favorable perinatal outcomes.

## 1. Introduction

Several studies [1–4] showed that fetal sleeping periods can lead to falsely nonreactive tests, therefore, increasing the risk of unnecessary obstetric intervention. Attempts to arouse the fetus while is sleeping or in a rest-activity cycle include a change in maternal position, physical activity, maternal glucose ingestion, sound stimulation, light stimulation, and manual fetal manipulation. However, the only stimuli that have consistently evoked responses in normal fetuses are acoustic and vibrotactile ones. Fetal vibroacoustic stimulation was first noted in 1947 by Bernard and Sontag [5] who observed that the fetal heart rate accelerated after acoustic stimulation [6] correlated fetal movements with fetal well-being. 

In modern obstetrics, fetal vibroacoustic stimulation uses an artificial larynx placed on the mother's abdomen over the fetal head region. This is expected to induce a startle reflex in the fetus, with subsequent fetal movement and FHR acceleration [7]. Many authors have observed an increase in the frequency and size of accelerations after vibroacoustic stimulation during low FHR [8–10]. It is hypothesized that FHR acceleration following VAS provides reassurance of fetal well-being, obviating the need for further intervention [11, 12]. Moreover, acoustic stimulation of the fetus has been suggested to improve the efficiency of antepartum fetal heart rate testing [13, 14]. Some authors in nonrandomized studies [15–17] have reported success using fetal vibroacoustic stimulation to improve the efficiency of antepartum fetal heart rate testing without changing the predictive reliability of the tests. Animal studies have revealed that fetal responses to extrauterine sound depend on the peripheral and central components of the fetus's auditory system. In lambs with bilateral cochlear ablation, no reactions towards vibroacoustic stimulation could be recorded, not even at high intensities, indicating that the auditory apparatus is necessary for fetal heart rate (FHR) and fetal movement (FM) responses in this animal model [18, 19]. Some authors have conducted studies to evaluate the existence of possible adverse effects caused by the VAS [20]. They concluded that stimulation with the electronic artificial larynx induced excessive fetal movements, a prolonged tachycardia, nonphysiological state changes, and a disorganisation and change in the distribution of fetal behavioural states, therefore, they did not recommend its use routinely. On the other hand, in more recent studies [21], vibroacoustic stimulation has been demonstrated not to cause any cochlea damage in the fetus, and they have shown that the intensity of sound and vibration are 4000 times less than in the amniotic fluid, compared to that produced in air. Moreover, an audiometric and tympanic screening performed on children who had been exposed to vibroacoustic stimulation in utero showed the absence of hearing loss [22].

Positive (reactive) VAS tests appeared comparable to standard reactive nonstress tests (NSTs) [23]. Perinatal outcomes following positive VAS appear to be similar to those associated with reactive NSTs, while testing time was shorter with VAS [24].

Since computerized cardiotocography (cCTG) provides an objective analysis of FHR, our aim of this study was to evaluate FHR before and after VAS, in both low- and high-risk pregnancies. Moreover, the cCTG parameters short-term variability (STV) and approximate entropy (ApEn) are considered more closely related to the actual well-being of the fetus, compared to the parameters detectable with the traditional reading [25]. Therefore, in the present study, we decided to consider especially them, in an attempt to provide an even more reliable evidence of fetal well-being. 

## 2. Materials and Methods

A retrospective study was performed in 563 pregnants referring to the “Federico II” University Medical School in Naples, between November 2007 and October 2010. The study was approved by ethics committee of the university and all participants gave their written informed consent.

Patients were selected according to some inclusion criteria: (a) availability of data from maternal medical history, ultrasound parameters, amniotic fluid production, Doppler velocimetry on umbilical cord; (b) computerized cardiotocographic (cCTG) analysis from antepartum FHR tracings before and after fetal vibroacoustic stimulation; (c) availability of data about the newborn baby (kind of delivery, sex, weight, Apgar score, malformations at birth, and the possible need of admission in neonatal intensive care). Moreover, each FHR tracing had to contain (a) a period of 10 minutes of continuous quiet before the stimulus was given and (b) a period of at least 10 minutes after the stimulus was given, both of acceptable quality, according to the analysis criteria [26]. Exclusion criteria for final analysis were (a) FHR tracings before the twenty-eighth week of pregnancy, for which the International Federation of Gynecology and Obstetrics does not provide any standard value for the cCTG parameters; (b) patient withdrawal from the study and/or unavailability of followup. Total population included 216 patients, divided in two groups: low- and high-risk pregnancies. “High-risk” pregnancy is generally thought of as the one in which the mother or the developing fetus has a condition that places one or both of them at a higher-than-normal risk for complications, either during the pregnancy (antepartum), during delivery (intrapartum), or following the birth (postpartum). Instead, low-risk pregnancy is defined for the absence of significant past medical or obstetric history and of fetal anomalies [27, 28]. The characteristics of the population of this study are shown in Tables 1 and 2. Pregnancy care was performed according to routine practice. All patients underwent the cCTG nonstress test in the same conditions, that is, in the same rooms, between 8.30 and 12.00 AM and with the women in semirecumbent position. Fetal heart rate was recorded using a computer-assisted equipment (Corometrics 170, General Electrics). Each FHR tracing was analyzed with the SEA 2CTG2 system that calculated the short term variability throughout this expression.

(1) $$\begin{array}{cc} \text{STV} & =\text{mean}\left[\left|T_{24}\left(i+1\right)-T_{24}\left(i\right)\right|\right]\\ & =\overset{23}{\underset{i=1}{\sum }}\frac{\left|T_{24}\left(i+1\right)-T_{24}\left(i\right)\right|}{23}. \end{array}$$

Approximate entropy (ApEn) derives from formulas suggested to estimate the Kolmogorov entropy of a process represented by a time series. It is a measurement of the conditional probability that two vectors that are close to each other for *m* points will remain close at the next point [29, 30].

**Figure 1:**
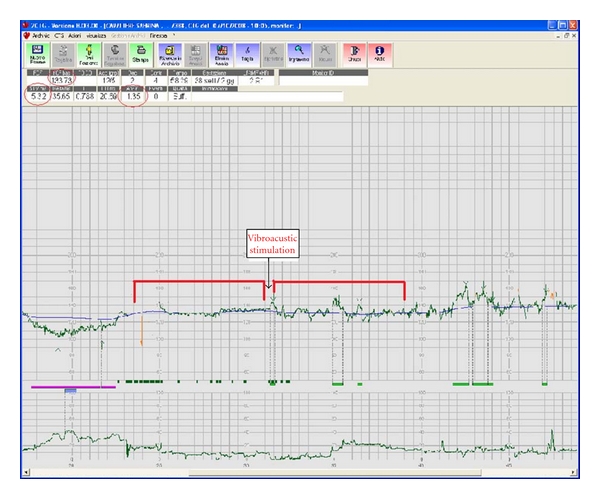
Example of computerized analysis of fetal heart rate tracing.

Baseline FHR, accelerations, decelerations, long-term irregularity (LTI), approximate entropy (ApEn), and fetal movements (FMs) were calculated for defined observational periods before and after VAS (Figure 1).

The cCTG parameters short term variability (STV) and the approximate entropy (ApEn) were analyzed in reference to the standard values proposed by Arduini and associates, for each gestational week [31, 32]. The aim of the study was to evaluate the changes in cCTG analysis before and after an external vibratory acoustic stimulus over the fetal vertex, by an artificial larynx, was produced. The stimulus consisted of 5 seconds of vibration applied to the maternal abdomen, while the fetus is sleeping, in accordance with the literature and the clinical indications [33]. Data were also investigated in relationship with the perinatal outcome. Adverse perinatal outcome included one or more of the following criteria: small for gestational age infant, fetal acidaemia at delivery, 5-minute Apgar score <7, respiratory distress syndrome, perinatal death, hypoxic ischemic encephalopathy, malformations, or other injuries requiring neonatal intensive care.

Statistical analysis was performed using chi-square test for independent samples, and the two-tailed Student's *t*-test. A *P* value < 0.05 was considered to be statistically significant.

## 3. Results

Fetal vibroacoustic stimulation was performed on 121 high-risk and 95 low-risk pregnancies, while their FHR parameters were monitored and recorded by cCTG analysis.

Significant fetal heart accelerations (*P* < 0.05) appeared 10 seconds after the stimulation both in low- and high-risk pregnancies. As many authors argued [34, 35], this event can be related to the sudden change of the fetal state from quiet sleep to activity/weakeness.

In each group of patients, FHR after VAS remained almost unmodified, as differences were not statistically significant (Tables 3 and 4). We considered positive a VAS test if the stimulation is follwed by the increase of STV and the decrease of ApEn. High-risk pregnant women showed an increase of STV in 76 cases (62.8%), a decrease of ApEn in 67 cases (55.37%), and a global positive test in 39 cases (32.23%), including 31 (38.7%) with a favorable perinatal outcome and 8 (20%) with an unfavorable outcome at birth.

In low-risk patients, we found an increase of STV in 72 cases (75.79%) and a decrease of ApEn in 49 cases (51.57%), with a positive VAS test in 36 cases (37.90%), including 32 (37.20%) with a favorable perinatal outcome and 4 (44.4%) with an unfavorable outcome. 50 babies (23.4%) showed adverse conditions at birth: 41 (33.88%) in high-risk pregnancies and 9 (9.47%) in low-risk pregnant women.

**Figure 2:**
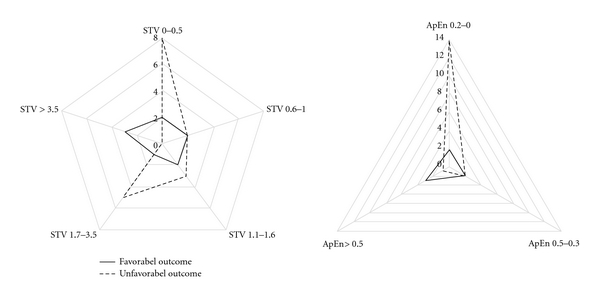
Radar graph representing the cutoff values found for short term variability (STV) and approximate entropy (ApEn).

Fetal movements significantly increased after VAS in both the groups (*P* = 0.001). Only in the high-risk pregnancies, the increase of STV and the decrease of ApEn after VAS were significantly associated with favorable perinatal outcomes. Moreover, chi-square test (*χ* ^2^) showed that only between 28th and 36th week of gestation and only when STV increased more than 3.5 ms and ApEn decreased more than 0.5, the relationship with the perinatal outcome was statistically significant (Table 5). The radar graph in Figure 2 summarizes the cutoff values found, offering an immediate visual evaluation about the different relationships between STV and ApEn with the perinatal outcome, respectively.

VAS predictive value was examined through the analysis of sensitivity and specificity. Sensitivity was found to be 37% in low-risk pregnancies, and 39% in high-risk pregnancies. Instead, specificity was 55% and 80%, respectively, in low- and high-risk pregnancies (Table 6).

## 4. Discussion

Vibroacoustic stimulation offers a unique opportunity to assess how the fetus responds to the external environment. Fetal VAS is commonly used for both antepartum and intrapartum testing. It is considered a simple and reliable prognostic evaluation of abnormal FHR detection. A metaanalysis performed by Skupski et al. [36] of reports on intrapartum fetal stimulation tests, analyzed different types of fetal stimulation, including scalp puncture for pH testing, the use of an Allis clamp to pinch the fetal scalp, digital stroking of the fetal scalp, and VAS. All the methods resulted similar. Moreover, methods like fetal scalp blood sampling, fetal electrocardiography, and fetal pulse oximetry are invasive, their benefit can be uncertain, and are not widely available or used routinely [37]. Fetal VAS is a noninvasive technique that does not involve puncture of the amniotic membrane, making it particularly useful in antepartum primary care. Ohel et al. [8] demonstrated that after vibroacoustic stimulation occurs, a statistically significant increase in basal FHR (115 bpm) during periods of low FHR reactivity and a statistically significant increase in the number of fetal movements happen. Bartnicki and Dudenhausen [38] argued that after an acoustic stimulus, fetal movements cause a partial occlusion of the umbilical cord that could be responsible for the reduced fetal pressure and acute hypoxia that would in turn lead to a reflex stimulation of the autonomous nervous system.

From the current literature, we know that changes in the beat-to-beat interval are mainly mediated by the regulation mechanisms and by the synergic action elicited by the autonomic nervous system (ANS) activity, through its sympathetic and parasympathetic branches. As the activities of the ANS are closely related with those of the central nervous system, and their contributions are usually quantified through parameters obtained from the spectral analysis of HRV signal [39, 40]. Therefore, the analysis of FHR signals of fetuses submitted to a vibroacoustic stimulation may provide information about the functional aspects of the autonomic nervous system and of the central nervous system, making possible diagnosis, prognosis, and monitoring of major pathologies of the central or peripheral nervous system.

Cardiotocography (CTG) provides a noninvasive monitoring of fetal heart rate (FHR). It is the most popular analysis method in clinical practice allowing the recording and the analysis of FHR, via ultrasonographic techniques. Indices obtained from the cardiotocographic FHR signals have been adopted to study the autonomic nervous system activity of the fetus during the intrauterine life both in physiologic and pathologic conditions [41]. Gonçalves et al. [42] showed a significant relationship between FHR linear and nonlinear parameters and fetal state, as linear indices increased significantly with rising fetal activity whereas the opposite occurred with nonlinear indices. D'Elia et al. [43] used the computerized cardiotocography to assess the fetal heart rate in healthy term fetuses subjected to VAS. He found a statistically significant increase not only for FM and number of FHR accelerations, but also in STV. Moreover, they showed that the response was influenced by fetus' behavioural state at the time of the stimulus.

Interestingly, a study [44] performing a linear and nonlinear fetal heart rate analysis in normal and acidemic fetuses before delivery showed that, with the progression of the labor, a significant increase in linear frequency domain indices and a significant decrease in nonlinear indices became evident. Bernardes et al. [45] hypothesized that fetal sex differences could affect the FHR response to VAS.

From our results, we can state that fetuses respond to vibroacoustic stimulation accelerating their FHR. However, the analysis of STV and ApEn after VAS shows that the difference is significant only in high-risk pregnancies. This result make these cCTG parameters a useful tool in the evaluation of fetal neuronal response. Especially noteworthy is the significant association of a positive VAS test with perinatal conditions in high-risk pregnancy, between the 28th and the 36th week of gestation. This result could be explained with the development of the auditory apparatus, which occurs in that period. Indeed, vibroacoustic stimulation elicits a neural response in the fetus, from the auditory sensory system to the central level, whose response is able to trigger the autonomic mechanisms responsible for the regulation of the FHR. The identification of cutoff values of STV and ApEn for the association with the perinatal outcome in high-risk pregnancies could provide useful tools for better understanding and management of pathophysiological neural development of the fetus. In addition, the sensitivity and the specificity of the results of the present study were analyzed. The low sensitivity values found, both in low- and high-risk pregnancies, show that Vas test was positive only in a small number of patients delivering healthy babies. On the other hand, the difference between the two groups, found in the specificity analysis, is very meaningful. The high percentage of specificity found for high-risk pregnancies (80%) suggested that a negative Vas test was observed in a large percentage of patients whose babies show negative outcome at birth, in contrast with the lower specificity (55%) found in low-risk pregnancies. This is to confirm the importance of the use of Vas test inside the cCTG analysis as a predictive tool of conditions at birth. Moreover, our findings can be seen as a confirmation of what Gonçalves et al. [44] hypothesized, that is, an increase in the autonomic nervous system activity and a decrease in the central nervous system activity, when a stress condition occurs (the final minutes of labor in Gonçalves' study and the vibroacoustic stimulation in the present study), even if we found differences between low- and high-risk pregnancies.

Differently from previous studies [4, 5, 8–10], our aim was not to study only the role of Vas in relationship with the modifications of fetal biophysical profile, but we wanted to analyze how its cardiotocographic effect relates with the perinatal outcome, in order to verify its predictive value. Moreover, the present study tried to find, for the first time, values of STV and ApEn from which it is possible to define a statistical significance of the relationship found. Further randomized trials are surely needed to determine reliable quantitative indices that could help a nearly recognition of central and peripheral pathologies. For this purpose, computerized cardiotocography analysis proves to be a reliable noninvasive method.

## Figures and Tables

**Table 1:** Patients' characteristics.

	Number	%
Gestational age at CTG recording (week):		
28–36 weeks 37–42 weeks	87 129	40.27 59.72
High-risk pregnancy	121	56
Low-risk pregnancy	95	44
Abnormal Doppler velocimetry on umbilical cord	10	4.63
Abnormal AFI, *n* (%):		
<5 cm	6	2.8
>25 cm	4	1.8

CTG, cardiotocogram; AFI, amniotic flux index.

**Table 2:** Babies' characteristics.

		Number	%
	SGA	17	7.8
Adverse perinatal outcome	5 min Apgar score <7	23	10.7
	RDS	2	0.93
	Perinatal death	2	0.93
	NICU admission	6	2.8

	Good perinatal outcome	166	76.85

SGA, small for gestational age; RDS, respiratory distress syndrome; NICU, neonatal intensive care unit.

**Table 3:** FHR parameters before and after VAS in low-risk pregnancies (*n* = 95).

	Before VAS	After VAS	P*
FHR	137.14	137.33	N.S.
FM	36	72	0.001
N° ACC	2.2	8.6	0.001
DEC	0.01	0.11	N.S.
STV	4.75	5.28	N.S.
ApEn	1.35	1.31	N.S.

Data are expressed as mean.

FHR, fetal heart rate; FM, fetal movements; ACC, accelerations; DEC, deceleration; STV, short-term variability; ApEn, approximate entropy; NS, not significant.

*Student's *t*-test, *according to the standard values proposed by Arduini.

**Table 4:** FHR parameters before and after VAS in high-risk pregnancies (*n* = 121).

	Before VAS	After Vas	P*
FHR	136.12	136.33	N.S.
FM	31	75	0.001
N° ACC	2.1	9.7	0.001
DEC	0.02	0.13	N.S.
STV	4.41	5.92	0.005
ApEn	1.36	1.22	0.005

Data are expressed as mean.

FHR, fetal heart rate; FM, fetal movements; ACC, accelerations; DEC, deceleration; STV, short-term variability; ApEn, approximate entropy; NS, not significant.

*Student's *t*-test.

**Table:** (a) Ranges of increase of STV (ms) after VAS

Perinatal outcome	0–0.5	0.6–1	1.1–1.6	1.7–3.5	>3.5
Favorable (number)	2	2	2	1	3
Unfavorable (number)	8	2	3	5	0

**Table:** (b) Ranges of decrease of ApEn after VAS

Perinatal outcome	0.2–0	0.5–0.3	>0.5
Favorable (number)	2	2	3
Unfavorable (number)	14	2	1

**Table:** (a) High-risk pregnancies

	Positive perinatal outcome	Negative perinatal outcome
Positive VAS Test	31	8
Negative VAS Test	49	33

Sensitivity = 31/(31 + 49) = 0,39 = 39%.

95% confidence interval = 0,39 ± 1,96 *√* 0,39 · (1 − 0,39)/(31 + 49) = 0,2831–0,4969.

Specificity = 33/(8 + 33) = 0,8 = 80%.

95% confidence interval = 0,8 ± 1,96 *√* 0,8 · (1 − 0,8)/(8 + 33)= 0,6776–0,9224.

**Table:** (b) Low-risk pregnancies

	Positive perinatal outcome	Negative perinatal outcome
Positive VAS Test	32	4
Negative VAS Test	54	5

Sensitivity = 32/(32 + 54) = 0,37 = 37%.

95% confidence interval = 0,37 ± 1,96 *√* 0,37 · (1 − 0,37)/(32 + 54) = 0,27–0,47.

Specificity = 5/(4 + 5) = 0,55 = 55%.

95% confidence interval = 0,55 ± 1,96 *√* 0,55 · (1− 0,55)/(4 + 5) = 0,23–0,87.
